# Markers of sulfadoxine–pyrimethamine resistance in Eastern Democratic Republic of Congo; implications for malaria chemoprevention

**DOI:** 10.1186/s12936-019-3057-7

**Published:** 2019-12-18

**Authors:** Marit van Lenthe, Renske van der Meulen, Maryvonne Lassovski, Adelaide Ouabo, Edwige Bakula, Colette Badio, Deogratias Cibenda, Lucy Okell, Erwan Piriou, Lynn Grignard, Kjerstin Lanke, Bhargavi Rao, Teun Bousema, Cally Roper

**Affiliations:** 1 Médecins Sans Frontières (MSF), Amsterdam, The Netherlands; 2 Radboud University Medical Centre, Nijmegen, The Netherlands; 3 London School of Hygiene and Tropical Medicine, London, UK; 4 MSF, London, UK; 5 Programme National de Lutte contre le Paludisme (PNLP) South Kivu, Bukavu, Democratic Republic of Congo; 6 MSF, North Kivu, Goma, Democratic Republic of Congo; 7 MSF, Bukavu, Democratic Republic of Congo; 8 Imperial College, London, UK

**Keywords:** Malaria, DRC, Sulfadoxine–pyrimethamine (SP), Chemoprophylaxis, *dhps*, *dhfr*, K540E, A581G, I164L, IPTi

## Abstract

**Background:**

Sulfadoxine–pyrimethamine (SP) is a cornerstone of malaria chemoprophylaxis and is considered for programmes in the Democratic Republic of Congo (DRC). However, SP efficacy is threatened by drug resistance, that is conferred by mutations in the *dhfr* and *dhps* genes. The World Health Organization has specified that intermittent preventive treatment for infants (IPTi) with *SP* should be implemented only if the prevalence of the *dhps* K540E mutation is under 50%. There are limited current data on the prevalence of resistance-conferring mutations available from Eastern DRC. The current study aimed to address this knowledge gap.

**Methods:**

Dried blood-spot samples were collected from clinically suspected malaria patients [outpatient department (OPD)] and pregnant women attending antenatal care (ANC) in four sites in North and South Kivu, DRC. Quantitative PCR (qPCR) was performed on samples from individuals with positive and with negative rapid diagnostic test (RDT) results. *Dhps* K450E and A581G and *dhfr* I164L were assessed by nested PCR followed by allele-specific primer extension and detection by multiplex bead-based assays.

**Results:**

Across populations, *Plasmodium falciparum* parasite prevalence was 47.9% (1160/2421) by RDT and 71.7 (1763/2421) by qPCR. Median parasite density measured by qPCR in RDT-negative qPCR-positive samples was very low with a median of 2.3 parasites/µL (IQR 0.5–25.2). Resistance genotyping was successfully performed in RDT-positive samples and RDT-negative/qPCR-positive samples with success rates of 86.2% (937/1086) and 55.5% (361/651), respectively. The presence of *dhps* K540E was high across sites (50.3–87.9%), with strong evidence for differences between sites (p < 0.001). *Dhps* A581G mutants were less prevalent (12.7–47.2%). The *dhfr* I164L mutation was found in one sample.

**Conclusions:**

The prevalence of the SP resistance marker *dhps* K540E exceeds 50% in all four study sites in North and South Kivu, DRC. K540E mutations regularly co-occurred with mutations in *dhps* A581G but not with the *dhfr* I164L mutation. The current results do not support implementation of IPTi with SP in the study area.

## Background

Despite a decline in cases and deaths over the past 20 years, malaria forms a major health problem in Africa. In the Democratic Republic of Congo (DRC) cases of malaria are rising, and the death toll is increasing [1]. In 2017 in DRC there was an estimated number of 25 million (15.7–36.8) cases of malaria and an estimated 46,800 (36,200–57,300) deaths. The majority of malaria cases in DRC are caused by *Plasmodium falciparum*, although *Plasmodium ovale* [2]*, Plasmodium malariae,* and *Plasmodium vivax* are present among symptomatic cases [3]. Médecins sans Frontières (MSF) has been working in DRC since 1991. The Operational Centre Amsterdam of MSF (MSF–OCA) has several projects in Eastern DRC, most notably in North and South Kivu. Both provinces are the scene of chronic and at times intense conflict, leading to frequent internal displacement, outbreaks of disease, sexual violence, malnutrition, and a collapse of healthcare services. MSF supports several large hospitals and health centres and has adopted a strategy of Integrated Community Case Management (ICCM). Within the ICCM programme, community members are trained to diagnose and treat malaria, diarrhoea and pneumonia in children under 5 years of age. MSF and the Ministry of Health distribute insecticide-treated bed nets in the community as well as targeted distribution to pregnant women, severe malaria patients and other specific groups. Additional interventions are implemented where supportive evidence warrants it, aiming to reduce the burden of malaria. These interventions include chemoprevention strategies that are delivered without the need for testing. Sulfadoxine–pyrimethamine (SP) is a cornerstone of malaria chemoprophylaxis across the African continent. Intermittent preventive treatment of malaria in pregnancy (IPTp) and intermittent preventive therapy for infants (IPTi) use SP alone, while seasonal malaria chemoprophylaxis (SMC) combines SP with amodiaquine. The efficacy of SP for these interventions is influenced by drug resistance levels in parasite populations [4] and the drug resistance levels are influenced by the use of SP in target populations [5, 6].

The genetic basis of SP resistance is well understood, and consequently molecular surveillance of resistance mutations underpins current policy on SP use in Africa. SP resistance occurs via the accumulation of mutations in the dihydrofolate reductase (*dhfr*) gene and in the dihydropteroate synthase (*dhps*) gene and these progressively reduce susceptibility to pyrimethamine and sulfadoxine, respectively. Infections harbouring triple mutant *dhfr*, i.e., a combination of N51I, C59R and S108N mutations, are common throughout Africa. When this triple mutation in *dhfr* is combined with double-mutant *dhps* (A437G and K540E) the risk of SP treatment failure is up to 75% [7, 8]. A ‘sextuple mutant’ genotype in which these five key mutations in *dhfr* and *dhps* gene are accompanied by *dhps* A581G is increasingly reported [9].

**Fig. 1:**
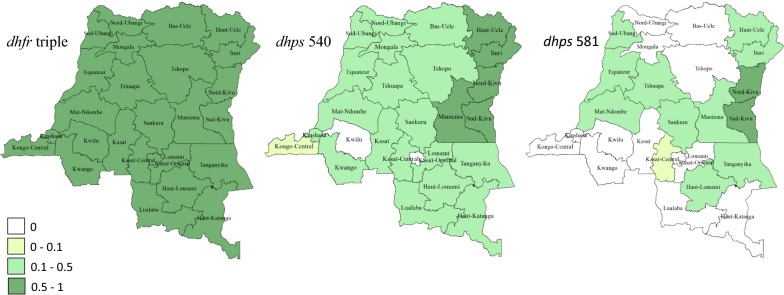
The prevalence of SP resistance markers. The prevalence of SP resistance markers in each of the 25 districts of the DRC 2013–2014 (redrawn from [25]). Administrative districts are shaded according to the prevalence of mutations. **a** The ‘*dhfr* triple’ containing N51I,C59R and S108N. **b** The *dhps* K540E. **c** The *dhps* A581G. *SP* sulphadoxine–pyrimethamine, *dhfr* dihydrofolate reductase, *dhps* dihydropteroate synthase

The World Health Organization (WHO) has specified that IPTi-SP should only be implemented in areas where the prevalence of the *dhps* K540E mutation (a proxy measure for the presence of all 5 key mutations) is under 50% [10]. Molecular surveillance reports up to 2016 have recorded prevalence of K540E exceeding the 50% threshold in 12 countries: most in East Africa (Sudan, Somalia, the Republic of the Congo, Tanzania, Malawi, Kenya, DRC, Mozambique, Ethiopia, Rwanda, Uganda, Zambia) [9], with relevant within-country variation. In the case of IPTp, SP continues to provide protection in pregnancy in all these areas except within restricted geographic areas where the *dhps* 581G is also present at high prevalence [4, 11–14]. One such area is confined to southwest Uganda, Rwanda and bordering areas of Eastern DRC. A study in 2013–2014 to survey small numbers of samples from all 25 districts of DRC found that prevalence of the sextuple mutant genotype is concentrated in North and South Kivu (Fig. 1), which are the districts bordering southwestern Uganda and Rwanda.

There are indications that the range and prevalence of the sextuple genotype is increasing [9]. This study aimed to update molecular surveillance data from North and South Kivu with more substantial sample sizes and examine the geographic heterogeneity in prevalence of 540E and 581G in order to determine if IPTi could be a preventive strategy used in MSF mission sites within these provinces of Eastern DRC.

## Methods

### Study area, subjects and sample collection

From August to September 2017, samples were collected in the context of MSF projects in two provinces: North Kivu and South Kivu. Each province provided samples from two study sites. The aim was to obtain a minimum of 250 samples with confirmed *P. falciparum* infection per study site from either pregnant women attending ANC or individuals attending the clinic with suspected malaria.

The sample size was based on the assumption of 50% prevalence of *dhps* K540E and the ability to estimate this expected proportion with 5% precision for each site. The study received ethical approval from the London School of Hygiene & Tropical Medicine (#11976), the Ethics Review Board of MSF (#1708) and the Commission Institutionnelle d’Ethique of the Catholic University of Bukavu (UCB/ClE/NC/005B/2017). Informed consent was obtained from all participants or their carers prior to participation. Young adolescents received information appropriate for their age before they were asked for consent.

Study participants were passively recruited at MSF clinics from pregnant women presenting for ANC and from patients presenting with signs/symptoms of malaria (axillary temperature ≥ 37.5 °C or history of fever). Malaria HRP2-test rapid diagnostic tests (RDTs) (SD BIOLINE Malaria Ag P.f, 05FK50; Gyeonggi-do, Republic of Korea) were performed to detect presence of *P. falciparum* antigen. Two blood spots were collected on filter papers (Whatman^**®**^ 903 Protein Saver Card; Maidstone, UK), air-dried, stored individually in zip-lock bags with desiccant at room temperature, and kept away from humidity, excessive heat and light. All dried blood spots (DBS) samples, whether RDT positive or negative, were shipped at ambient temperature to Radboud University Medical Centre, The Netherlands, for further processing.

### Laboratory procedures

DNA was extracted from two 3-mm DBS punches using Chelex® 100 chelatin resin, according to the manufacturer’s recommendations, and using a filter (WHA77002808 ALDRICH Whatman^®^ UNIFILTER^®^ plates) to avoid Chelex in the final DNA sample. DNA from both RDT positive and negative samples was tested using a qPCR assay targeting the *P. falciparum*-specific 18S gene [15]. This assay was originally optimized for use on whole blood samples (and not filter paper samples). For the current project, parasite density was quantified using in vitro trendline material on filter paper (Whatman^**®**^ 903 Protein Saver Card; Maidstone, UK) to have the same conditions for test samples and trendlines. For all qPCR positive samples, nested PCR amplification was done for *dhps;* for a sub-set of samples where *dhps* amplification was successful, *dhfr* was also amplified (n = 387). Primers were based on previous publications [16, 17] and are presented in Table 1. PCR conditions were 94 °C × 3 min, followed by 40 rounds of 94 °C × 1 min → 51 °C × 2 min → 72 °C × 1 min, and a final round of 72 °C for 10 min. Samples were evaluated by gel electrophoresis prior to further analysis.

**Table 1:** Primer sequences

Gene	Primer/probe	Sequence
*Pf18S*	18S forward	5′GTAATTGGAATGATAGGAATTTACAAGGT 3′
*Pf18S*	18S reverse	5′TCAACTACGAACGTTTTAACTGCAAC 3′
*Pf18S*	18S probe	6FAM-AACAATTGGAGGGCAAG–MGBNFQ
*Dhps*	Outer forward	5′ GATTCTTTTTCAGATGGAGG 3′
*Dhps*	Outer reverse	5′ TTCCTCATGTAATTCATCTGA 3′
*Dhps*	Inner forward	5′ AACCTAAACGTGCTGTTCAA 3′
*Dhps*	Inner reverse	5′ AATTGTGTGATTTGTCCACAA 3′
*Dhfr*	Outer forward	5′ TGATGGAACAAGTCTGC 3′
*Dhfr*	Outer reverse	5′ ACTTTGTTTATTTCCATTCA 3′
*Dhfr*	Inner forward	5′ TGTGCATGTTGTAAGGTTGA 3′
*Dhfr*	Inner reverse	5′ GATACTCATTTTCATTTATTTCTGGA 3′
*Dhps*	540 K	5′ *ATTAAACAACTCTTAACTACACAA*GGAAATCCACATACAATGGATA 3′
*Dhps*	540 E	5′ *CTTTCTTAATACATTACAACATAC*GAAATCCACATACAATGGATG 3′
*Dhps*	581 A	5′ *CATAAATCTTCTCATTCTAACAAA*TTGATATTGGATTAGGATTTGC 3′
*Dhps*	581 G	5′ *CAAACAAACATTCAAATATCAATC*TTGATATTGGATTAGGATTTGG 3′
*Dhfr*	164 I	5′ *CATAAATCTTCTCATTCTAACAAA*GAAATTAAATTACTATAAATGTTTTATTA 3′
*Dhfr*	164 L	5′ *CAAACAAACATTCAAATATCAATC*GAAATTAAATTACTATAAATGTTTTATTT 3′

Listed are primer sequences used in qPCR to confirm *P. falciparum* infection and quantify parasite density (Pf18S), nested PCR for amplifying Dhps and Dhfr for ASPE reactions for hybridisation with different alleles. Sequence highlighted in italic is the Luminex tag sequence

*qPCR* quantitative polymerase chain reaction, *Dhps* dihydropteroate synthase; *Dhfr* dihydrofolate reductase, *ASPE* Allele Specific Primer Extension

*dhps* K450E and A581G and *dhfr* I164L were assessed by nested PCR followed by allele specific primer extension (ASPE) and detection on a Luminex instrument (MAGPIX^®^; Luminex Corp) with xPONENT® 4.2 software. Briefly, amplified DNA was incubated with ExoSAPit for 30 min at 37 °C, followed by 15 min at 80 °C. Samples were then assembled with the ASPE master mix [250 nM each of ASPE probes and 200 µM Biotin-14-dCTP (Invitrogen, UK)] using primers from Table 1 and incubated 30 cycles (30 s 90 °C, 1 min 53.5 °C, 2 min 72 °C). Utilizing different TAGs on different ASPE primers, multiple magnetic bead sets could be hybridized to the ASPE reactions. For each reaction, 1 μL of each bead set was used. After two washes with Tm buffer (0.2 M NaCl, 0.1 M Tris, 0.08% TritonX-100, pH 8), Tm buffer + 0.1% BSA + 2.5 μg/mL Streptavidin-R-phycoerythrin (SAPE) was added to each sample. Samples were analysed at 37 °C on the Luminex analyser according to manufacturer’s instructions.

### Statistical analysis

Statistical analyses were performed in STATA version 15.0 (StataCorp; College Station, TX, USA). Data were typically presented per site and separately for sample donors recruited at clinics with suspected malaria (suspected clinical cases) or pregnant women attending antenatal care (ANC participants). Age was categorized in 5 categories (< 2 years, 2–4 years, 5–9 years, 10–14 years, ≥ 15 years). Parasite density estimates were compared between RDT-positive and -negative individuals by non-parametric Wilcoxon rank-sum test; median values with 25th and 75th percentile (IQR) were presented. Parasite prevalence estimates were given for suspected clinical cases and ANC participants separately, adjusting 95% CI for the cluster-design of data collection using the survey (svy) command in STATA. Genotyping results were presented per site and separately for suspected clinical cases and ANC participants.

## Results

**Fig. 2:**
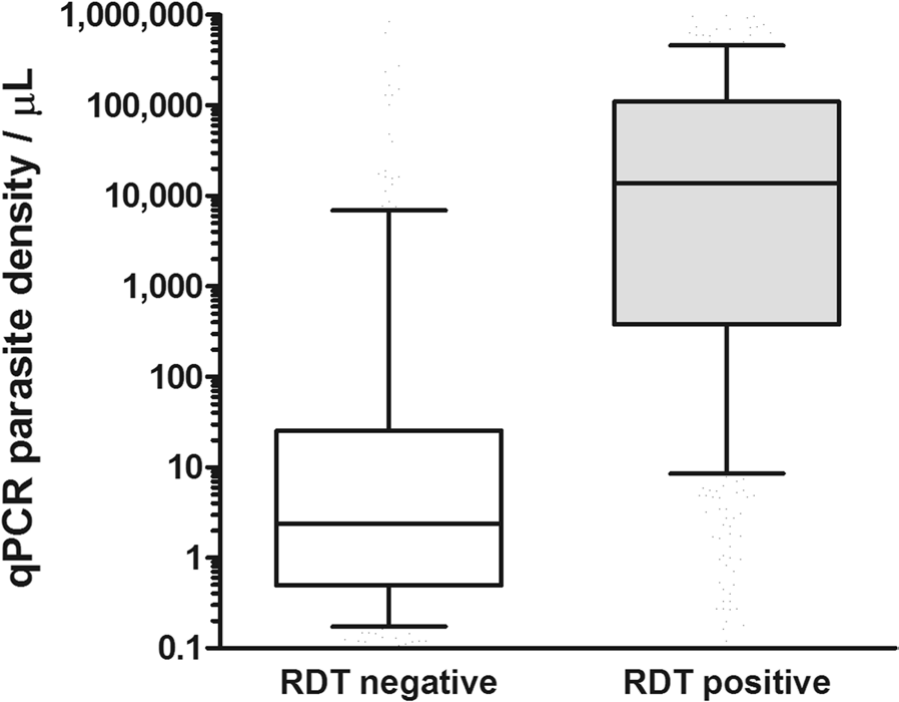
Parasite density estimated by 18S quantitative PCR in RDT negative and positive individuals. *qPCR* quantitative polymerase chain reaction, *RDT* rapid diagnostic test; SD Bioline. The box plots present median and interquartile range densities; whiskers indicate the 10th and 90th percentile

RDT and qPCR results were available for a total of 2421 individuals from the four study sites; data were unavailable for 83 additional participants because of missing blood spot samples (n = 8), errors in identification codes (n = 3) and laboratory errors/assay failures (n = 72). The majority of participants with successfully analysed samples were collected at clinics from suspected clinical malaria cases (78.8%, 1907/2421), the remainder coming from ANC participants (Table 2). Across study sites, parasite prevalence by RDT was higher in suspected malaria cases (45.4%, 95% CI 31.7–59.9) compared to ANC participants (23.2; 95% CI 6.8–55.6%) (p = 0.026), taking into account a study site as primary sampling unit. Similarly, parasite prevalence by qPCR was higher in suspected malaria cases (78.0%, 95% CI 72.1–82.9) compared to ANC participants (48.4; 95% CI 25.9–71.6%) (p = 0.029). Individuals who were RDT-positive were significantly more likely to be qPCR-positive compared to RDT-negative individuals (OR 13.8, 95% CI 3.49–54.4, p = 0.009). Nevertheless, 47.8–67.4% of RDT-negative suspected clinical cases and 25.0–52.0% of RDT-negative ANC participants were found to be qPCR-positive in the four study sites. The estimated median parasite density by qPCR in RDT-positive individuals was 13,608 parasites/µL (IQR 384–108,600) compared to 2.3 parasites/µL (IQR 0.5–25.2) in RDT-negative individuals (Fig. 2; p < 0.001).

**Table 2:** Characteristics of study participants

	Baraka	Kimbi	Walikale	Mweso
Suspected clinical malaria	500	415	662	330
Antenatal visits	169	151	73	121
Age, % (n)				
< 2 years	19.9 (133)	21.8 (123)	14.3 (105)	12.0 (54)
2–4 years	20.9 (140)	30.3 (171)	17.1 (125)	13.1 (59)
5–9 years	17.2 (115)	2.1 (12)	17.9 (131)	13.3 (60)
10–14 years	6.4 (43)	1.6 (9)	14.6 (107)	11.3 (51)
≥ 15 years	35.6 (238)	44.2 (249)	36.2 (265)	50.3 (227)
Parasite prevalence RDT, % (n/N)				
Clinic	47.2 (236/500)	50.6 (210/415)	64.1 (424/662)	51.8 (171/330)
ANC	9.5 (16/169)	32.5 (49/151)	50.7 (37/73)	14.1 (17/121)
Parasite prevalence qPCR, % (n/N)				
Clinic	81.2 (406/500)	74.9 (311/415)	79.8 (528/662)	73.3 (242/330)
ANC	41.4 (70/169)	64.9 (98/151)	57.5 (42/73)	32.2 (39/121)

*Clinic* samples from individuals attending the clinic with suspected clinical malaria, *ANC* samples from individuals attending antenatal care, *RDT* rapid diagnostic test SD Bioline, *qPCR* quantitative polymerase chain reaction, *n* absolute number, *N* total sample size per location

**Fig. 3:**
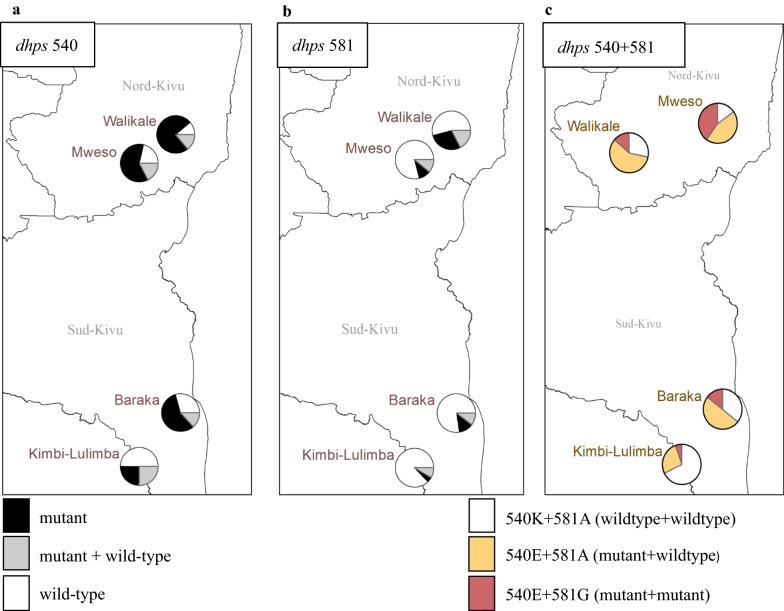
Prevalence of dhps K540E and 581G mutations at the 4 MSF sites. The number of infections carrying pure mutant, mixed (mutant + wildtype) or pure wildtype infections are shown for dhps K540E (**a**), A581G (**b**) m and the haplotypes of dhps 540 and 581 at each site (**c**). *dhps* dihydropteroate synthase

Amplification of the *dhps* gene was successful for 86.2% (937/1086) of RDT-positive samples and 55.5% (361/651) of RDT-negative/qPCR-positive samples. All *dhps* positive samples were genotyped for *dhps* K540E and A581G and categorized as having a pure or mixed genotype for each locus (Table 3, Additional file 1: Table S1). The presence of *dhps* K540E, either as pure mutant or as mixed mutant/wild-type infections, exceeded 50% in all study sites, with strong evidence for differences between sites (p < 0.001) (Fig. 3). *dhps* A581G mutants were less prevalent than *dhps* K540E; only in Mweso approximately half of all infections carried the 581G as pure mutant or mixed wild-type/mutant. Again, there was strong evidence for differences in the prevalence of the *dhps* A581G mutation between sites (p < 0.001). The *dhps* A581G mutation almost exclusively occurred in the presence of the *dhps* K540E mutation: 100.0% (143/143) of all *dhps* A581G pure mutant and 96.2 (128/133) of all mixed wildtype/mutant infections had concurrent mutations in *dhps* K540E.

**Table 3:** The prevalence of mutations in *Dhps 540* and *Dhps 581* in the different study sites

	Baraka	Kimbi	Walikale	Mweso
	Clinic	Clinic	Clinic	Clinic
N	302	352	365	174
Dhps 540, % (n)				
Wildtype	29.8 (90)	49.7 (175)	21.1 (77)	12.1 (21)
Pure mutant	56.6 (171)	25.0 (88)	61.9 (226)	75.3 (131)
Mixed	13.6 (41)	25.3 (89)	17.0 (62)	12.6 (22)

N	314	360	377	178
Dhps 581, % (n)				
Wildtype	78.0 (245)	87.2 (314)	78.8 (297)	52.8 (94)
Pure mutant	12.1 (38)	4.4 (16)	10.1 (38)	30.3 (54)
Mixed	9.9 (31)	8.3 (30)	11.1 (42)	16.9 (30)

*N* total sample size per location, *n* absolute number, *Dhps* dihydropteroate synthase

The *dhfr* gene was successfully amplified in 94.9% (333/351) of randomly selected samples (Baraka: n = 78; Kimbi: n = 91; Walikale: n = 96; Mweso: n = 68). Among these samples, the *dhfr* I164L mutation was found in only one sample (0.3%, 1/333). The sample was collected from an RDT-confirmed clinical malaria case from Walikale and was wild type for *dhps* K540E and A581G.

## Discussion

High numbers of malaria cases continue to pose an intense burden to DRC and to the individuals affected. Due to conflict and unrest in the country, there is limited current information available on parasite resistance to anti-malarial drugs. This knowledge gap hinders the informed use of anti-malarial interventions. The current study aimed to provide estimates of molecular markers conferring SP resistance in four settings in an area of chronic conflict in Eastern DRC.

Parasite prevalence among suspected malaria cases was uniformly high in the study settings. When sensitive qPCR was used to detect *P. falciparum* parasites, parasite prevalence was 70–80% among suspected malaria cases and 32–65% among women attending ANC visits. These findings confirm the high malaria burden in this part of DRC [18]. The higher prevalence of parasites by qPCR compared to RDT corroborates an increasing body of literature on low-density infections that are present in all age groups and across all endemicities in Africa and elsewhere [19, 20]. Parasite densities among RDT-negative qPCR-positive individuals were very low on average, below the threshold density typically detected by microscopy or RDT [19]. Whilst this suggests adequate performance of RDTs in the study settings in symptomatic cases, deletions in histidine-rich protein (HRP2), as described in DRC by Parr et al. [21] will be examined in the current dataset in future studies. It is currently unclear whether parasite densities below the threshold for detection by RDT contribute to acute clinical disease in semi-immune individuals, although low-density infections have been associated with haemolysis, inflammation, co-infection with invasive bacterial disease, cognitive impairment [22], and ongoing transmission of parasites to mosquitoes [23]. At present, it may be assumed that RDT-negative individuals presenting with suspected malaria had fever due to causes other than *P. falciparum* infection [24].

A high level of the *dhps K540E* mutation was observed in this region and this is consistent with studies that reviewed and mapped all publicly available data on 540E in Africa [9, 25, 26]. At all sites, the prevalence of *dhps K540E* mutant exceeds 50%, the threshold level defined by WHO to determine whether or not to introduce IPTi [27]. The highest prevalence of 540E was in the eastern sites (Mweso and Baraka), closest to the border with southwest Uganda and western Rwanda, a region previously identified as a hotspot with a high level of SP resistance [25, 26]. This is consistent with the east–west gradient in the spatial distribution of the *dhps* mutations across DRC observed by Aydemir et al. [28] and summarized in Fig. 1. Okell et al. showed that resistance patterns are similar for sites up to 300 km apart [9] and Mweso is < 300 km from resistant hotspots in southwest Uganda [29, 30].

This study is consistent with a sequential build-up of resistance markers for SP. The A581G mutation almost exclusively occurred in the presence of K540E mutations [31]. The prevalence of I164L mutation on the *dhfr* gene was still very low, detected in only one of 333 samples. Future monitoring is needed to examine if the prevalence of this mutation is increasing and will have implications for SP efficacy. A relevant limitation of the current study is that samples were collected on one time-point only and solely collected at clinical facilities in suspected malaria cases and women attending ANC (routine screening of all pregnant women, including asymptomatically infected individuals). Some of the suspected malaria cases with measured fever and qPCR-or RDT-detected infections may have had fever due to causes other than malaria [24] and may have inappropriately received the diagnosis of clinical malaria. Whilst this misclassification is of no concern for the current objectives, estimating the prevalence of resistance markers among health facility attendees, it complicates a direct comparison of resistance in clinical versus asymptomatic infections.

The findings in this study are disappointing from a programmatic perspective. The study was initiated with the hope that IPTi could be introduced in the region to reduce morbidity and mortality. However, with resistance markers well above the threshold of 50% (for K540E) this cannot be supported. Although WHO still recommends IPTp with SP in areas with high level of resistance to SP, based on studies in Tanzania and Malawi [32], the current findings of high prevalence of SP resistance among the target population suggest that the efficacy of IPTp in this setting should continue to be monitored [33] and alternative drugs such as dihydroartemisinin–piperaquine, mefloquine or doxycycline may be worth considering [34–36].

The current findings highlight significant challenges for the use of SP for chemoprevention in this region in DRC. It is therefore of utmost importance that other interventions, including vector control and prompt access to diagnosis and treatment, are implemented to the highest attainable standards to reduce exposure and prevent severe disease.

## Conclusion

In the study areas of intense malaria transmission in North and South Kivu, the prevalence of the SP resistance marker *dhps* K540E was above 50% in all study sites. K540E mutations regularly coincided with mutations in *dhps* A581G but not with the *dhfr* I164L mutation. The current results do not support implementation of IPTi. Given the high morbidity and mortality from malaria in DRC, alternative approaches are needed for prevention and early treatment of malaria.

## Supplementary information

**Additional file 1: Table S1.** The prevalence of mutations in *Dhps 540* and *Dhps 581* for sample donors with suspected clinical malaria and donors attending antenatal care.

## Data Availability

Data are available on request in accordance with MSF’s data sharing policy at data.sharing@msf.org.

## Abbreviations

ANC: antenatal care
ASPE: allele specific primer extension
DBS: dried blood spots
*dhfr*: dihydrofolate reductase
*dhps*: dihydropteroate synthase
DNA: deoxyribonucleic acid
DRC: Democratic Republic of Congo
HRP2: histidine-rich protein 2
ICCM: Integrated Community Case Management
IPT*i*-*SP*: intermittent preventive therapy for infants
IPTp: intermittent preventive treatment of malaria in pregnancy
IQR: interquartile range
RDT: malaria HRP2-test rapid diagnostic test
MSF: Médecins sans Frontières
MSF–OCA: Médecins sans Frontières–Operational Centre Amsterdam
OPD: outpatient department
qPCR: quantitative polymerase chain reaction
SMC: seasonal malaria chemoprophylaxis
SP: sulfadoxine–pyrimethamine
WHO: World Health Organization
