# Supplementary material for: Markers of sulfadoxine–pyrimethamine resistance in Eastern Democratic Republic of Congo; implications for malaria chemoprevention
Source: Malar J. 2019 Dec 18;18:430. doi: 10.1186/s12936-019-3057-7 (PMC6921399; doi:10.1186/s12936-019-3057-7)
Supplement: Supplementary file 1 — Additional file 1: Table S1. The prevalence of mutations in Dhps 540 and Dhps 581 for sample donors with suspected clinical malaria and donors attending antenatal care. [file 12936_2019_3057_MOESM1_ESM.docx]

Table S1. The prevalence of mutations in *Dhps 540* and *Dhps 581* for sample donors with suspected clinical malaria and donors attending antenatal care.

|  |  | Baraka |  | Kimbi |  | Walikale |  | Mweso |  |
| --- | --- | --- | --- | --- | --- | --- | --- | --- | --- |
|  |  | Clinic | ANC | Clinic | ANC | Clinic | ANC | Clinic | ANC |
| N |  | 253 | 49 | 270 | 82 | 335 | 30 | 160 | 14 |
| Dhps 540, % (n) | Wildtype | 27.7 (70) | 40.8 (20) | 46.7 (126) | 59.8 (49) | 21.5 (72) | 16.7 (5) | 10.0 (16) | 35.7 (5) |
|  | Pure mutant | 58.5 (148) | 46.9 (23) | 25.6 (69) | 23.2 (19) | 62.1 (208) | 60.0 (18) | 79.4 (127) | 28.6 (4) |
|  | Mixed | 13.8 (35) | 12.2 (6) | 27.8 (75) | 17.1 (14) | 16.4 (55) | 23.3 (7) | 10.6 (17) | 35.7 (5) |
| N |  | 264 | 50 | 274 | 86 | 347 | 30 | 163 | 15 |
| Dhps 581, % (n) | Wildtype | 78.0 (206) | 78.0 (39) | 87.2 (239) | 87.2 (75) | 78.4 (272) | 83.3 (25) | 50.9 (83) | 73.3 (11) |
|  | Pure mutant | 12.1 (32) | 12.0 (6) | 4.0 (11) | 5.8 (5) | 10.4 (36) | 6.7 (2) | 31.9 (52) | 13.3 (2) |
|  | Mixed | 9.9 (26) | 10.0 (5) | 8.8 (24) | 7.0 (6) | 11.2 (39) | 10.0 (3) | 17.2 (28) | 13.3 (2) |
